# Dacomitinib for Advanced Non-small Cell Lung Cancer Patients Harboring Major Uncommon EGFR Alterations: A Dual-Center, Single-Arm, Ambispective Cohort Study in China

**DOI:** 10.3389/fphar.2022.919652

**Published:** 2022-06-13

**Authors:** Hong-Shuai Li, Guang-Jian Yang, Yi Cai, Jun-Ling Li, Hai-Yan Xu, Tao Zhang, Li-Qiang Zhou, Yu-Ying Wang, Jin-Liang Wang, Xing-Sheng Hu, Xiang Yan, Yan Wang

**Affiliations:** ^1^ Department of Medical Oncology, National Cancer Center/National Clinical Research Center for Cancer/Cancer Hospital, Chinese Academy of Medical Sciences and Peking Union Medical College, Beijing, China; ^2^ Department of Respiratory Medicine, Shandong Cancer Hospital and Institute, Shandong First Medical University and Shandong Academy of Medical Sciences, Ji’nan, China; ^3^ Independent Researcher, Ellicott City, MD, United States; ^4^ Department of Oncology, The Fifth Medical Center, Chinese PLA General Hospital, Beijing, China

**Keywords:** dacomitinib, non-small cell lung cancer, major uncommon EGFR mutations, efficacy, safety

## Abstract

**Objective:** Dacomitinib has been approved for non-small-cell lung cancer (NSCLC) patients harboring classical epidermal growth factor receptor (*EGFR*) mutations; however, clinical evidence of its activity on major uncommon *EGFR* mutations is currently limited.

**Materials and methods:** This was a dual-center, single-arm, ambispective cohort study in China. Patients with histologically confirmed metastatic or recurrent NSCLC harboring major uncommon *EGFR* mutations were eligible for the study. The objective response rate and disease control rate were determined by RECIST 1.1 every 1–2 months. Adverse events were assessed by CTCAE 5.0.

**Results:** In total, 32 NSCLC patients were enrolled between July 2020 and January 2022, and 18 (56.3%) patients received dacomitinib as first-line therapy. Median age was 64 years, and 20 (62.5%) were female. The mutations identified were G719X (*n* = 24; 75%), followed by L861X (*n* = 10; 31.3%), and S768I (*n* = 8; 25%). In the first-line setting, 72.2% of patients (13/18) had a confirmed partial response and 100% (18/18) had disease control, and the median progression-free survival (PFS) and overall survival (OS) were unreached. In the whole cohort, 56.3% of patients (18/32) had a confirmed partial response and 90.6% (29/32) had disease control, and the median PFS was 10.3 months (95% confidence interval, 6.1–14.5) and the median OS was 36.5 months. Except for one case not available for brain re-evaluation, control of the intracranial metastases was observed in 13 patients (13/14, 92.9%). No grade 4–5 adverse events (AEs) occurred, but all patients had grade 1–2 AEs, and 12.5% (4/32) patients required a dosage reduction due to intolerable AEs.

**Conclusions:** Dacomitinib demonstrated favorable activity with manageable toxicity in patients with NSCLC harboring major uncommon *EGFR* mutations.

## 1 Introduction

The in-frame deletion in exon 19 (19del; 49–72%) and one nucleotide substitution within codon 858 of exon 21 (L858R; 28–43%) are two predominant epidermal growth factor receptor (*EGFR*) alterations in non-small cell lung cancer (NSCLC), called “common mutations” or “classic mutations” (Wang and Li, 2019). Patients with NSCLC harboring these tyrosine kinase inhibitor (TKI)-sensitive mutations respond remarkably to *EGFR*-TKIs. Other *EGFR* mutations (10–20%), also called “uncommon mutations,” include any mutations other than 19del or L858R. These include exon 18 point mutations (e.g., E709X and G719X), exon 19 insertion mutations, exon 20 insertion mutations, exon 20 point mutations such as S768I, and exon 21 point mutations (e.g., L861Q) (Baek et al., 2015; Cho et al., 2020; Gristina et al., 2020; Wu et al., 2020; Passaro et al., 2021). These uncommon mutations represent a subset of NSCLC with poorer prognosis and a less favorable *EGFR*-TKI efficacy (Chiu et al., 2015a). Among them, exon 20 insertion mutations are the most prevalent and are largely insensitive to *EGFR*-TKIs (Remon et al., 2020; Huang et al., 2021). However, major uncommon mutations (including G719X, S768I, and L861Q) have also been found to be sensitive to *EGFR*-TKIs, especially the second-generation (2G) TKI afatinib. Recent National Comprehensive Cancer Network guidelines recommend afatinib as the preferred TKI for NSCLC harboring major uncommon mutations.

Dacomitinib is a potent, irreversible, highly selective 2G *EGFR*-TKI that inhibits signaling from both heterodimers and homodimers of all members of the human EGFR family. The significantly superior progression-free survival (PFS) benefit of dacomitinib over gefitinib in the ARCHER 1050 trial provided the basis for its use as a standard first-line option in *EGFR*-positive advanced NSCLC (Wu et al., 2017). A recent case series study of 14 patients has shown that dacomitinib is also effective against central nervous system (CNS) metastasis in *EGFR*-positive NSCLC (Peng et al., 2021). In addition, dacomitinib has potential applications in patients harboring uncommon mutations (Jänne et al., 2011; Reckamp et al., 2014; Kris et al., 2015; Nishino et al., 2018). In our previous study, we presented the later-line efficacy of dacomitinib on patients with NSCLC harboring uncommon *EGFR* mutations in a relatively small scale (*n* = 11) retrospectively, and the findings indicated a promising activity of dacomitinib (Li et al., 2022).

In this ambispective cohort study, we aimed to evaluate the efficacy and safety of dacomitinib in patients with NSCLC harboring major uncommon *EGFR* mutations in a relatively large scale.

## 2 Materials and Methods

### 2.1 Study Design and Patients

Chinese patients with NSCLC harboring major uncommon *EGFR* mutations treated with dacomitinib between July 2020 and January 2022 were ambispectively recruited from the Chinese PLA General Hospital and the National Cancer Center/National Clinical Research Center for Cancer/Cancer Hospital, Chinese Academy of Medical Sciences and Peking Union Medical College. The inclusion criteria were as follows: 1) Cytologically or histologically confirmed diagnosis of metastatic or recurrent NSCLC; 2) Harbor major uncommon *EGFR* mutations (including G719X, L861X, and S768I); and 3) Cell-free DNA from plasma, cerebrospinal fluid, or pleural effusion samples or tumor tissue before dacomitinib initiation were analyzed using the next-generation sequencing (NGS) method, which was conducted by our hospital and third-party qualified inspection institutions accredited by the College of American Pathologists.

### 2.2 Treatment and Efficacy/Toxicity Evaluation

All patients were treated with dacomitinib monotherapy. Unlike the standard dacomitinib dosing regimen of 45 mg used in the ARCHER 1050 study, in this real-world study, the starting dose was determined by the physician based on the patient’s condition. In general, the starting dose was 45 mg for patients with an Eastern Cooperative Oncology Group performance status (ECOG PS) of 0 and weight equal or greater than 60 kg; 30 mg for patients with an ECOG PS of one and weight less than 60 kg; 30 mg for patients with an ECOG PS of one and weight equal or greater than 60 kg, with an increase to 45 mg if well-tolerated by the patient; and 15 mg for patients with an ECOG PS of equal or greater than 2. A stepwise dose reduction of dacomitinib was conducted according to drug tolerance based on the above principles.

Imaging evaluation, including chest and abdomen computed tomography and brain magnetic resonance imaging (MRI), was performed every 1–2 months after drug administration. The objective tumor response was determined according to the Response Evaluation Criteria in Solid Tumors (version 1.1) guidelines. Objective response was categorized as complete response (CR) or partial response (PR), and disease control was categorized as CR, PR, or stable disease (SD). Toxicity was evaluated per the Common Terminology Criteria for Adverse Events version 5.0.

### 2.3 Statistical Analysis

For patient’s characteristics, categorical variables were reported as absolute numbers and percentages. Survival curves were constructed by the Kaplan-Meier method (Log-rank test). The data cutoff date was January 15, 2022, when the disease status of the patients was determined. PFS was defined as the period from dacomitinib initiation to disease progression or any-cause death. Overall survival (OS) was defined as the period from dacomitinib initiation to any-cause death. Patients who were lost to follow-up were censored, and the last determinable time of survival was used as the time of termination of follow-up. All statistical analyses were performed and all pictures were created using GraphPad Prism nine software (GraphPad Software, San Diego, CA, United States). *p* values < 0.05 denoted statistically significant differences.

## 3 Results

### 3.1 Baseline Patient Characteristics

**FIGURE 1:**
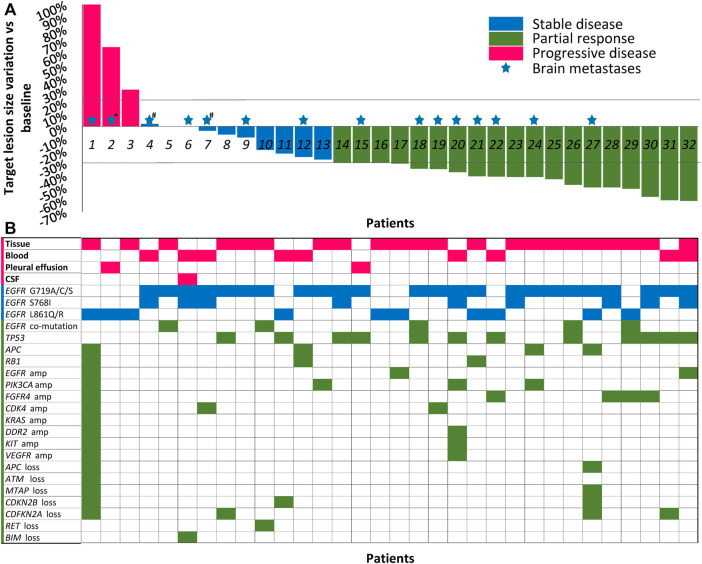
Best shrinkage of target lesion size and corresponding mutation profile by patient (*N* = 32). **(A)** Treatment responses of dacomitinib in all 32 patients are shown in the waterfall plot. Patients with compound mutations and brain metastases were marked with colored pentagrams. *Brain metastases not evaluated due to patient’s poor condition. #The patients also received whole brain radiotherapy before dacomitinib initiation. Dashed lines represent 20% progression (progressive disease) and 30% tumor regression (partial response). **(B)** The corresponding mutation profile of each patient was displayed under the waterfall plot. CSF, cerebrospinal fluid; amp, amplification.

A total of 32 patients with *EGFR*-mutant NSCLC treated with dacomitinib were enrolled. The clinical, demographic, pathological, and molecular characteristics of the cohort are shown in Table 1. More than 60% of the patients were women and never-smokers. The median age was 64 years. Overall, 15 patients (46.9%) had one or more nodules in the brain, and two patients (P4, P6) of them also had leptomeningeal metastasis (Figure 1A). Dacomitinib was applied as first-line treatment in more than half (18, 56.3%) of patients, and the median application line was 1 (range, 1–6). Most patients (65.5%) had an ECOG PS of 1. More than 60% of patients received 30 mg of dacomitinib as the starting dose.

**TABLE 1:** Baseline characteristics (N = 32).

Characteristics	*n* (%)
**Median age** year/(range)	64 (41–83)
**Gender**	
Female	20 (62.5) 12 (37.5)
Male	
**Smoking history**	
Current smoker	10 (31.3) 3 (9.4) 19 (59.4)
Former smoker	
Never smoker	
**Histology**	
AC	31 (96.9) 1 (3.1)
ASC	
**Stage**	
Relapsed	9 (28.1) 23 (71.9)
IV	
**Brain metastases**	
Yes	15 (46.9) 17 (53.1)
No	
**Total tumor burden**	
<3 metastatic organs	24 (75.0) 8 (25.0)
≥3 metastatic organs	
**ECOG PS**	
0	8 (25.0)
1	20 (62.5)
2	4 (12.5)
Mutation subtype*	
G719X	24 (75)
G719A/C	13
G719A+L861Q	2
G719A+L861R	1
G719A+S768I	2
G719C+S768I	4
G719S+S768I	2
L861X	10 (31.3)
L861Q	7
L861R	1
L861Q+G719A	2
S768I	8 (25)
S768I+G719A	2
S768I+G719C	4
S768I+G719S	2
**Dacomitinib application line**	Median (range): 1 (1–6)
1	18 (56.3)
2	9 (28.1)
≥3	5 (15.6)
**Dacomitinib starting dose**	
45 mg	9 (28.1)
30 mg	21 (65.6)
15 mg	2 (6.3)

AC, adenocarcinoma; ASC, adenosquamous carcinoma; ECOG PS, Eastern Cooperative Oncology Group performance status.

* Uncommon mutation categories overlap with compound mutations, so each patient might belong to more than one group.

### 3.2 Genetic Profiling

Cell-free DNA from plasma, cerebrospinal fluid, or pleural effusion samples or tumor tissue before dacomitinib initiation were analyzed utilizing the NGS method. Figure 1B shows the details of the major uncommon *EGFR* mutations and accompanying mutations. For specific mutation subtypes, 24 patients harbored G719X; 10 patients, L861X; eight patients, S768I (Table 1). G719A and L861Q were the two most frequent single uncommon mutations, and 11 patients had compound mutations, with G719C + S768I being the predominant (36.4%, 4/11). The most common concomitant mutations were detected in tumor protein p53 (*TP53*) (37.5%), cyclin-dependent kinase inhibitor 2A (12.5%), phosphatidylinositol-4,5-bisphosphate 3-kinase catalytic subunit alpha (12.5%), adenomatous polyposis coli (12.5%), cyclin-dependent kinase inhibitor 2B (9.4%), RB transcriptional corepressor 1 (9.4%), *EGFR* amplification (9.4%), fibroblast growth factor receptor 4 (9.4%), and cyclin-dependent kinase 4 (9.4%) genes (Figure 1B).

### 3.3 Treatment Modality and Efficacy/Toxicity Evaluation

All the enrolled patients received dacomitinib monotherapy. Nine patients received a dacomitinib starting dose of 45 mg once daily, while 21 patients received 30 mg, and two patients received 15 mg once daily until disease progression or intolerable toxicity. The specific dosing regimen was described in the **Methods** section.

**FIGURE 2:**
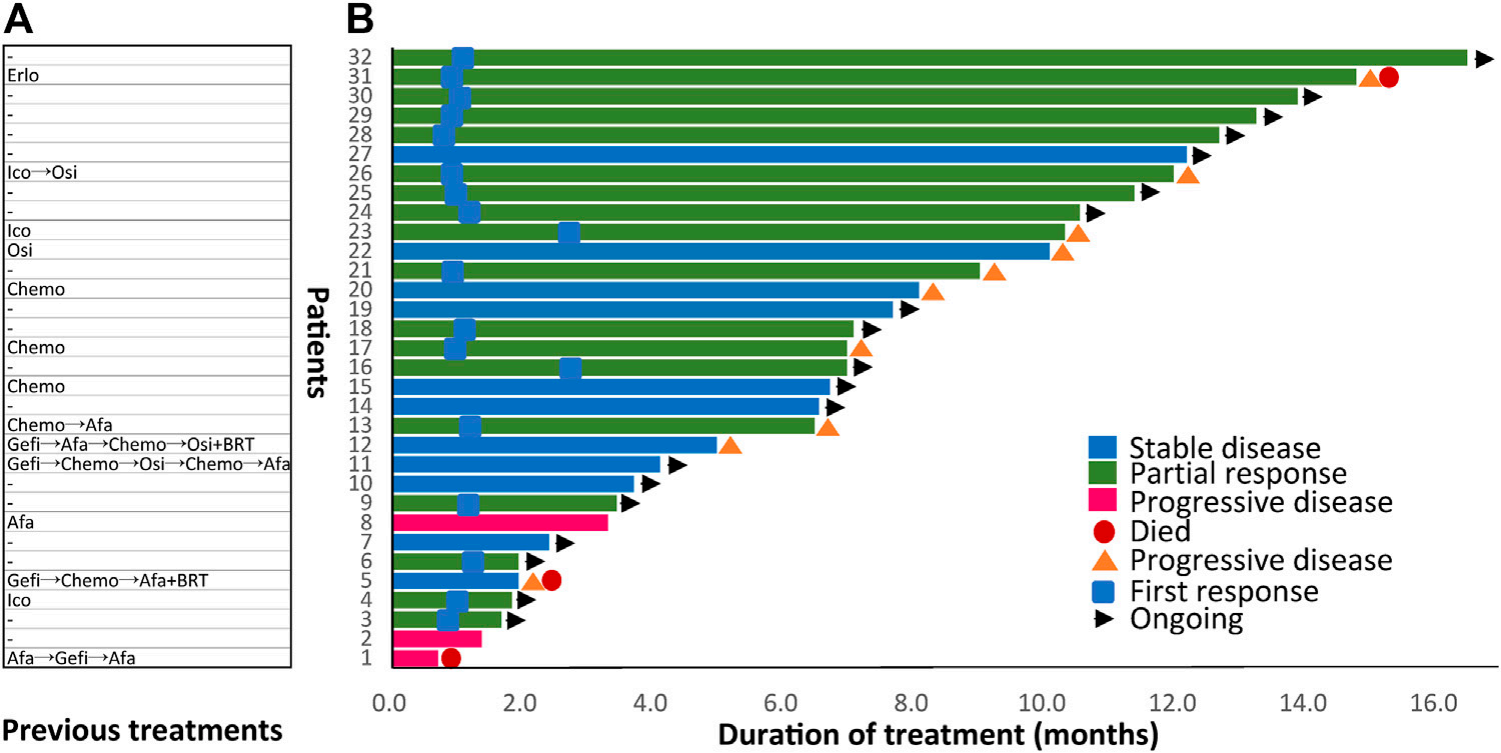
Previous treatments before dacomitinib initiation and duration of dacomitinib treatment by patient (*N* = 32). **(A)** Treatment history of each patient was displayed. **(B)** Duration of dacomitinib treatment of each patient was demonstrated correspondingly. Afa, afatinib; Gefi, gefitinib; Erlo, erlotinib; Ico, icotinib; Osi, osimertinib; Chemo, chemotherapy; BRT, whole brain radiotherapy.

In the overall cohort, 18 (56.3%) patients had PR, 11 (34.4%) had SD, and another three (9.4%) patients had *de novo* resistance to dacomitinib with PD as the best response **(** Figure 1A). Previous treatments before dacomitinib were shown in Figure 2A. The objective response rate (ORR) was 56.3% (18/32), and the disease control rate (DCR) was 90.6% (29/32). The median follow-up duration was 11.4 months (95% confidence interval [CI]: 7.4–15.4). The PFS was mature in 14 patients, and the tumors in 18 patients remained under control (Figure 2B).

**FIGURE 3:**
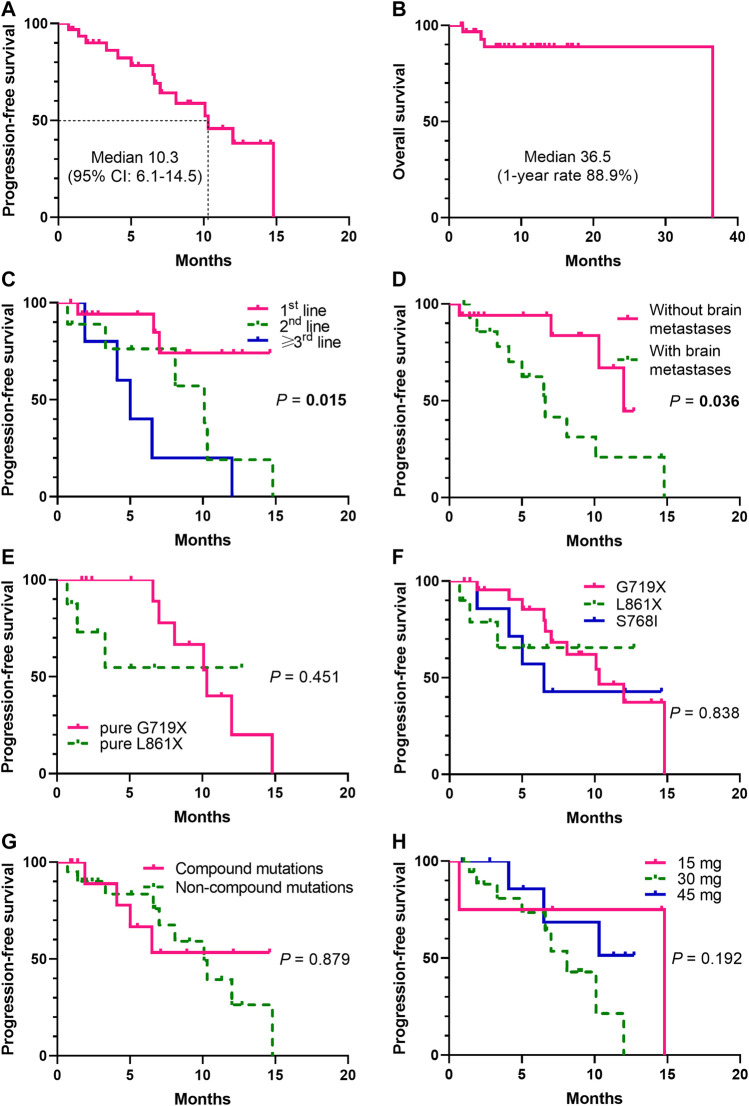
Kaplan-Meier analysis of progression-free survival (PFS) and overall survival (OS) (*N*= 32). The Kaplan-Meier curves were shown for PFS (Figure 3A) and OS (Figure 3B). Significant difference was observed (*p* = 0.015) in the median PFS (mPFS) of patients treated with dacomitinib in different lines (Figure 3C). Significant unfavorable effect of brain metastases on PFS (*p* = 0.036) (Figure 3D) was shown. Patients harboring different mutation subtypes demonstrated different mPFS (Figures 3E,F), though no significant difference was observed. No significant difference between compound mutations and non-compound mutations groups was found (*p* = 0.879) (Figure 3G). Patients received different dacomitinib doses showed no different mPFS (*p* = 0.192) (Figure 3H).

At the data cutoff date, the median PFS was 10.3 months (95% CI, 6.1–14.5) (Figure 3A), and median OS was 36.5 months (Figure 3B). Swimming plots of different mutation subgroups (Supplementary Figure S1A) and dose subgroups (Supplementary Figure S1A) were also generated. Subgroup analysis by mutation subtypes showed differences in response rates, with the L861X subgroup demonstrating the worst ORR (L861X *vs* G719X *vs* S768I: 44.4% *vs* 56.5% *vs* 62.5%) and DCR (66.7% *vs* 100% *vs* 100%). However, there was no significant difference in the ORR (*p* = 0.737) (Table 2).

**TABLE 2:** Treatment responses and prognosis of different mutation subgroups.

Mutation Subgroups	n	ORR (%)	P* Value	DCR (%)	P* Value	PFS (months)	*P* ^*#*^ Value
**Overall cohort**			0.737		**0.004**		0.830
*G719X (n=24)*		56.5		100		10.3	
G719A/C	13						
G719A/C/S+S768I	8						
G719A+L861Q/R	3						
*L861X (n=10)*		44.4		66.7		NR	
L861Q	7						
L861Q+G719A	2						
L861R	1						
*S768I (n=8)*		62.5		100		6.5	
S768I+G719A/C/S	8						
**First-line cohort**			0.325		0.269		0.665
*G719X (n=13)*		66.7		100		NR	
G719A/C	7						
G719C/S+S768I	4						
G719A+L861Q	2						
*L861X (n=7)*		50		83.3		NR	
L861Q	4						
L861Q+G719A	2						
L861R	1						
*S768I (n=4)*		100		100		NR	
S768I+G719C/S	4						

ORR, objective response rate; DCR, disease control rate; PFS, progression-free survival; NR, not reached. * *p-value* was calculated using the chi-squared test. ^#^ *p-values* were calculated using the log-rank test. Uncommon mutation categories overlap with compound mutations, so each patient might belong to more than one group.

Bold font indicates a statistically significant difference.

#### 3.3.1 First-Line Therapy Setting

Among 18 (56.3%) prospectively enrolled patients who received dacomitinib as the first-line therapy, after at least one radiologic review, 5 (27.8%) patients showed SD and 13 (72.2%) showed PR, achieving an ORR of 72.2% and a DCR of 100%. The median PFS was not reached (NR) (Figure 3C). Specifically, compared with the other patients with the G719A mutation, the third patient (P5) (Figure 1A) did not experience any shrinkage of the tumor lesion after treatment, and NGS testing suggested S899N and D1083V co-mutations of *EGFR*; however, the significance of these co-mutations is currently unknown.

#### 3.3.2 Later-Line Therapy Setting

In total, 14 (43.8%) retrospectively enrolled patients received dacomitinib as later-line therapy and previous treatments of each patient were displayed in Figure 2A. Five patients received dacomitinib after progression from first-line 2G TKI afatinib treatment (”2+2 mode”). Among them, two patients had PD, two patients had SD, and one patient achieved PR. Three patients were given dacomitinib after progression from third-generation TKI (”3+2 mode”) without T790M. Among them, two patients achieved SD and one patient developed PR. The PFS of the three patients was 5, 10.1, and 12 months, respectively.

#### 3.3.3 Brain Metastases Setting

**FIGURE 4:**
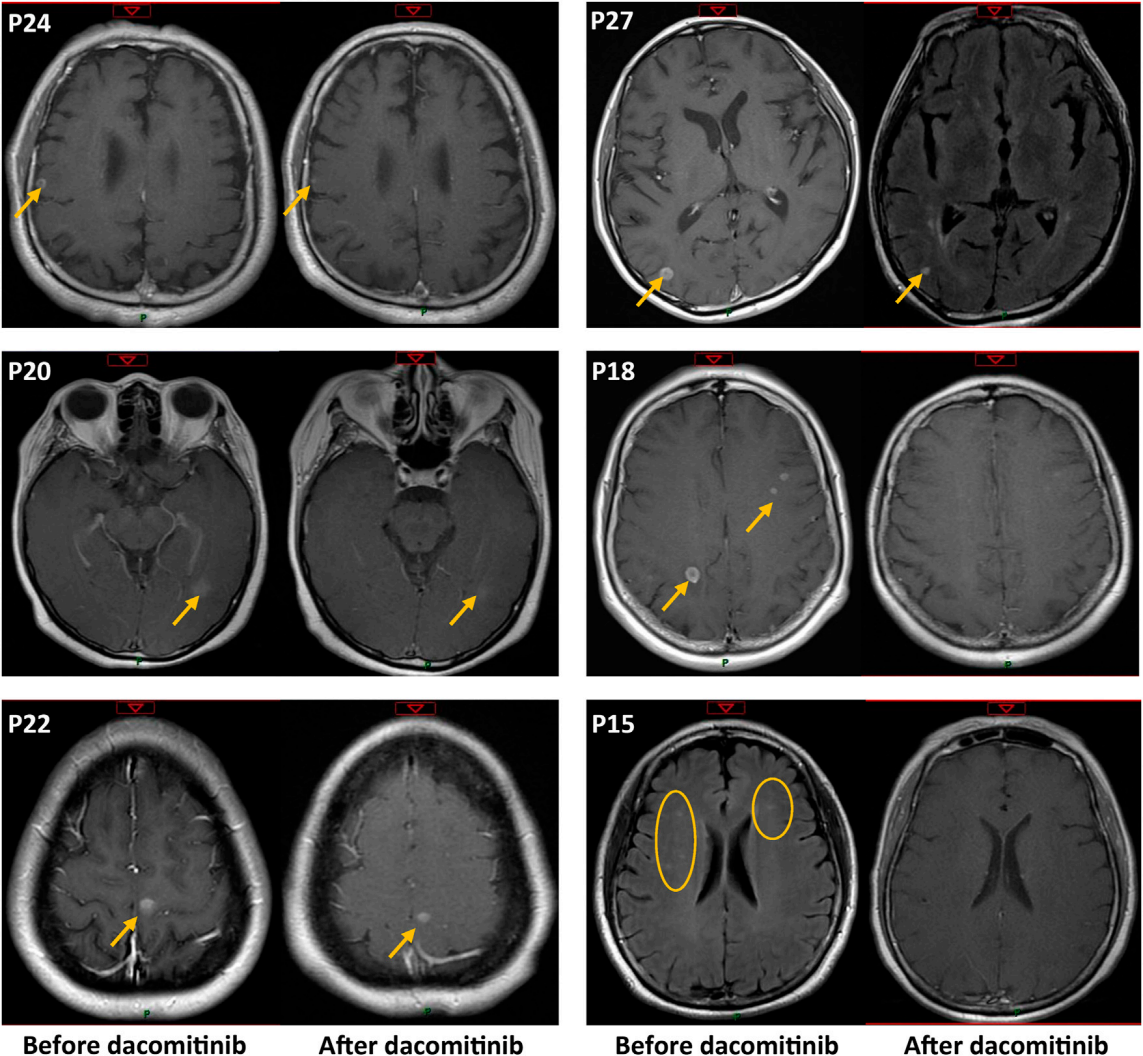
Enhanced MRI confirmed intracranial metastasis control before and after dacomitinib treatment in six representative patients. The patient number is consistent with Figure 1 but not Figure 2.

A total of 15 patients were diagnosed with brain parenchymal metastases before dacomitinib treatment, and 2 (P4, P6) of them also had concurrent leptomeningeal metastases (Figure 1A). Seven patients had symptoms associated with brain lesions, including dizziness, headache, and ambiopia, while the remaining eight patients had asymptomatic brain lesions. Two patients received whole brain radiotherapy before dacomitinib initiation. Except for one patient who was unable to undergo a brain examination due to obvious chest progression and poor ECOG PS, enhanced MRI confirmed disease control in 13 of the 14 patients (92.9%) (Figure 4). Five symptomatic patients experienced symptom relief. In addition, among the 10 patients with brain metastases who developed disease progression, four patients (40%) progressed again due to brain metastases.

In the overall cohort, the most common adverse events (AEs) were rash (86.7%), diarrhea (80%), and oral mucositis (60%). Three patients required a dosage reduction from 45 mg to 30 mg or 15 mg, one patient required a dosage reduction from 30 to 15 mg due to intolerable grade 3 diarrhea or rash, and two patients required a dosage escalation from 30 to 45 mg owing to good tolerance. All patients completed dose adjustment within 4 weeks. All patients (100%) experienced grade 1–2 AEs, but no grade 4–5 treatment-emergent AEs occurred (Table 3).

**TABLE 3:** Treatment-emergent AEs (N = 32).

AEs	G1	G2	G3
Diarrhea	20 (62.5)	4 (12.5)	2 (6.3)
Rash	17 (53.1)	9 (28.1)	2 (6.3)
Oral mucositis	13 (40.6)	5 (15.6)	2 (6.3)
Dry skin	9 (28.1)	3 (9.4)	1 (3.1)
Paronychia	5 (15.6)	3 (9.4)	0
Nausea	1 (3.1)	1 (3.1)	0
Hemorrhinia	1 (3.1)	0	0

Data are presented as n (%). AEs, adverse events. No grade 4–5 treatment-emergent AEs were observed.

### 3.4 Survival Analysis

The median PFS (mPFS) was significantly among patients treated with dacomitinib in the first-, second-, and third-line setting (NR vs 10.1 *vs* 5 months, *p* = 0.015) (Figure 3C). Brain metastases significantly affected PFS (HR = 2.997, 95% CI: 1.047–8.583; *p* = 0.036) (Figure 3D). The mPFS was different among the mutation subtypes pure G719X and pure L861X, although the difference did not reach statistical significance (10.3 *vs* NR; *p* = 0.451) (Figure 3E). The mPFS was also different among the mutation subtypes G719X, L861X, and S768I, although the difference did not reach statistical significance (10.3 *vs* NR *vs* 6.5; *p* = 0.838) (Figure 3F). There were also no significant differences between the compound and non-compound mutation subgroups (*p* = 0.879) (Figure 3G). In addition, the mPFS did not significantly differ according to the dacomitinib dose (*p* = 0.192) (Figure 3H). Survival comparisons according to age, gender, smoking history, ECOG PS, total tumor burden, and *TP53* co-mutation status showed no significant differences (Supplementary Figure S2).

## 4 Discussion

There have been few studies on the efficacy and safety of dacomitinib for uncommon *EGFR* mutations (Lau et al., 2019; Lavacchi et al., 2019; Wang and Li, 2019). In our previous study, we retrospectively presented the later-line efficacy of dacomitinib on 11 patients with NSCLC harboring uncommon *EGFR* mutations, and the findings indicated a promising activity of dacomitinib (Li et al., 2022). Further, in this study of 32 Chinese patients with NSCLC harboring major uncommon *EGFR* mutations treated with dacomitinib, 14 patients (46.7%) showed disease progression within a median follow-up duration of 11.4 months. Particularly, in the first-line setting, the patients obtained an ORR of 72.2% and a DCR of 100%, and the median PFS was unreached and the median OS was 36.5 months.

Common *EGFR* mutations are highly responsive to different types of *EGFR*-TKIs. However, uncommon mutations (especially exon 20 insertions) are less sensitive, with less satisfactory response rates and survival in previous studies (Wu et al., 2011; Watanabe et al., 2014; Baek et al., 2015; Chiu et al., 2015b; Kuiper et al., 2016; Krawczyk et al., 2017). Nevertheless, major uncommon *EGFR* mutations, including L861Q, G719X, and S768I, are still sensitive to first-generation (1G) *EGFR*-TKIs (gefitinib/erlotinib), with ORRs ranging from 41.6% to 53.8%; DCR, 62.9%–86.7%; mPFS, 2.2 months–7.7 months; and median OS (mOS), 11.9 months–19.0 months (Wu et al., 2011; Chiu et al., 2015b; Kuiper et al., 2016; Krawczyk et al., 2017). However, evidence suggests that 2G TKIs are more favorable in patients with NSCLC harboring uncommon mutations. Wu et al. (Wu et al., 2020) demonstrated that for patients with major uncommon *EGFR* mutations, the mPFS of afatinib was significantly longer than that of gefitinib and erlotinib. A combined post-hoc analysis of three LUX-Lung trials demonstrated that for the most frequent uncommon mutations (i.e., G719X, L861Q, and S768I), the ORRs were 77.8%, 56.3%, and 100%, and the mPFS were 13.8 months, 8.2 months, and 14.7 months, respectively (Yang et al., 2015).

Based on these promising results, afatinib was approved by the Food and Drug Administration in 2018 for lung cancer with L861Q, G719X, and S768I mutations. Nevertheless, efforts to explore more effective drugs for uncommon mutations have continued. In the KCSG-LU15-09 trial conducted by Cho et al. (Cho et al., 2020), osimertinib achieved an ORR of 53%, 78%, 38% and an mPFS of 8.2 months, 15.2 months, and 12.3 months in 32 patients (mostly first-line and all TKI-naïve) harboring major uncommon *EGFR* mutations of G719X, L861Q, and S768I, respectively. Dacomitinib is another 2G TKI that is an irreversible pan-human epidermal growth factor receptor TKI. Despite its potential, data on its usefulness in the treatment of NSCLC patients with major uncommon *EGFR* mutations are rare (1). In the current study, dacomitinib in the first-line setting achieved an ORR of 72.2% and a DCR of 100%. These values are close to the post-hoc analysis data for the 2G TKI afatinib from the LUX-Lung trial (24). Specifically, the ORRs for different mutation subtypes (G719X *vs* L861X *vs* S768I) were 66.7%, 50%, and 100%, respectively, which are also similar to the data reported by Yang et al. Collectively, these findings indicate that, compared to osimertinib, 2G TKIs yield a better treatment response for G719X and S768I; however, they have a worse therapeutic efficacy for L861X, consistent with the findings by Robichaux et al. (Robichaux et al., 2021).

In the new structure-based classification developed by Robichaux et al., G719X and S768I are categorized as the “P-loop C-helix compressing” type, where changes in the orientation of the P-loop could alter the position of TKI stabilization points, tilting the indole ring of osimertinib away from the P-loop and destabilizing drug binding. It is known that 2G TKIs do not interact with the P-loop of EGFR and maintain interaction points in the hydrophobic cleft, thus retaining full effectiveness for G719X and S768I, as supported by patient-derived xenograft model experiments. However, the L861Q mutation, categorized as the “classical-like” type, is distal from the drug-binding pocket and has lower impact on the overall structure of EGFR than the wild-type. In this mutation, osimertinib has better efficacy than 2G TKIs and is thus worthy of further research. The current study involved a higher proportion of females and never smokers, and this trend is similar to that seen in patients harboring common mutations. This is in contrast to previous studies on uncommon *EGFR* mutations (Wang et al., 2013; Chiu et al., 2015a; Tu et al., 2017; Yamada et al., 2020). For specific mutation subtypes, G719X (40.6%) and L861Q (21.9%) were the two most frequent single uncommon mutations. Overall, 34.4% of patients (11/32) had compound mutations, and G719X + S768I was the most common compound mutation (81.8%, 9/11), consistent with the mutation profiles reported by Yamada et al. (Yamada et al., 2020) and by Brindel et al. (Brindel et al., 2020).

Patients with compound uncommon mutations have better tumor response and prognosis than patients with single uncommon mutations (Brindel et al., 2020; Yamada et al., 2020; Yang et al., 2020; Passaro et al., 2021; Tan et al., 2021). In the study by Yang et al., afatinib yielded better anti-tumor activity against compound uncommon mutations than against major uncommon mutations in both TKI-naïve patients and TKI-pretreated patients (Yang et al., 2020). However, we found no significant difference in the ORR and mPFS between compound mutation and non-compound mutation groups. We speculate that this may be due to differences in ethnicity, study size, and mutation-type distribution. A recent case series by Peng et al. (Peng et al., 2021) showed that dacomitinib has potent efficacy against CNS metastasis in *EGFR*-positive NSCLC, with an ORR of 92.9% and a DCR of 100%. One of the patients with brain metastases carrying G719A mutation also achieved PR, indicating the potential efficacy of dacomitinib for NSCLC harboring uncommon mutations with brain metastases. In our study, disease control of brain metastatic lesions was confirmed in 13 patients (13/14, 92.9%). In addition, 5/8 (62.5%) patients with symptomatic brain metastatic experienced symptom relief, supporting the efficacy of dacomitinib for patients harboring uncommon mutations with brain metastases.

Interestingly, five patients who progressed with 2G TKI afatinib were treated with dacomitinib. In the OLCSG trial 1403, afatinib re-administration for sensitive *EGFR*-mutant NSCLC without T790M after resistance to 1G or 2G *EGFR*-TKIs yielded modest activity, with an ORR and DCR of 17% and 84%, respectively. However, no successful rechallenge with dacomitinib after afatinib progression has been reported to date, especially in patients harboring uncommon mutations. Masuda et al. (Masuda et al., 2020) reported long-term survival of NSCLC patients harboring concomitant G719C and S768I mutations who received afatinib rechallenge, indicating the potential of 2G TKI rechallenge. In our study, of the five patients treated with 2G afatinib followed by dacomitinib, two patients developed PD, two achieved SD, and one achieved PR, suggesting the possibility dacomitinib rechallenge after afatinib progression. However, additional clinical data are required to explore and confirm this strategy.

In the ARCHER 1050 study (Wu et al., 2017), dacomitinib treatment had to be discontinued in 7% of the patients due to toxic effects, and dose reductions due to intolerable AEs were required in 66% of the patients. However, Cheng et al. (Cheng et al., 2021) reported that the OS benefit was maintained in patients who had a stepwise dose reduction of dacomitinib (from 45 mg/day to 30 mg/day or 15 mg/day). Based on data from previous clinical trials, we adjusted the dose according to the patient’s physical status, weight, and comorbidities to ensure that the patient tolerated the AEs as soon as possible. No patient required treatment discontinuation due to serious AEs, and most patients were able to adapt to the therapy within 4 weeks. In addition, only 12.5% (4/32) of the patients required a dosage reduction due to intolerable AEs. However, due to the limited study scale, no definite conclusions could be made on whether different doses affect the efficacy and survival benefit of dacomitinib.

This study had some limitations. The sample size was small, and the patients were only enrolled from two medical centers in China. Therefore, the possibility of selection bias, which may have resulted in potentially compromised results, could not be ruled out. Thus, the results should be interpreted cautiously. Furthermore, the mechanisms of dacomitinib resistance have not been investigated. Our findings need to be confirmed in prospective clinical trials.

## 5 Conclusion

This ambiospective cohort study shows that dacomitinib has potential efficacy in advanced NSCLC patients harboring major uncommon *EGFR* mutations. In addition, dacomitinib has favorable efficacy for brain metastases and has a good safety profile. Dacomitinib may be a new paradigm and expectation for patients with major uncommon *EGFR* mutations, and more prospective data are warranted.

## Data Availability

The original contributions presented in the study are included in the article/Supplementary Material, further inquiries can be directed to the corresponding authors.
