# Supplementary figures and images for: Dacomitinib for Advanced Non-small Cell Lung Cancer Patients Harboring Major Uncommon EGFR Alterations: A Dual-Center, Single-Arm, Ambispective Cohort Study in China
Source: Front Pharmacol. 2022 Jun 13;13:919652. doi: 10.3389/fphar.2022.919652 (PMC9234690; doi:10.3389/fphar.2022.919652)

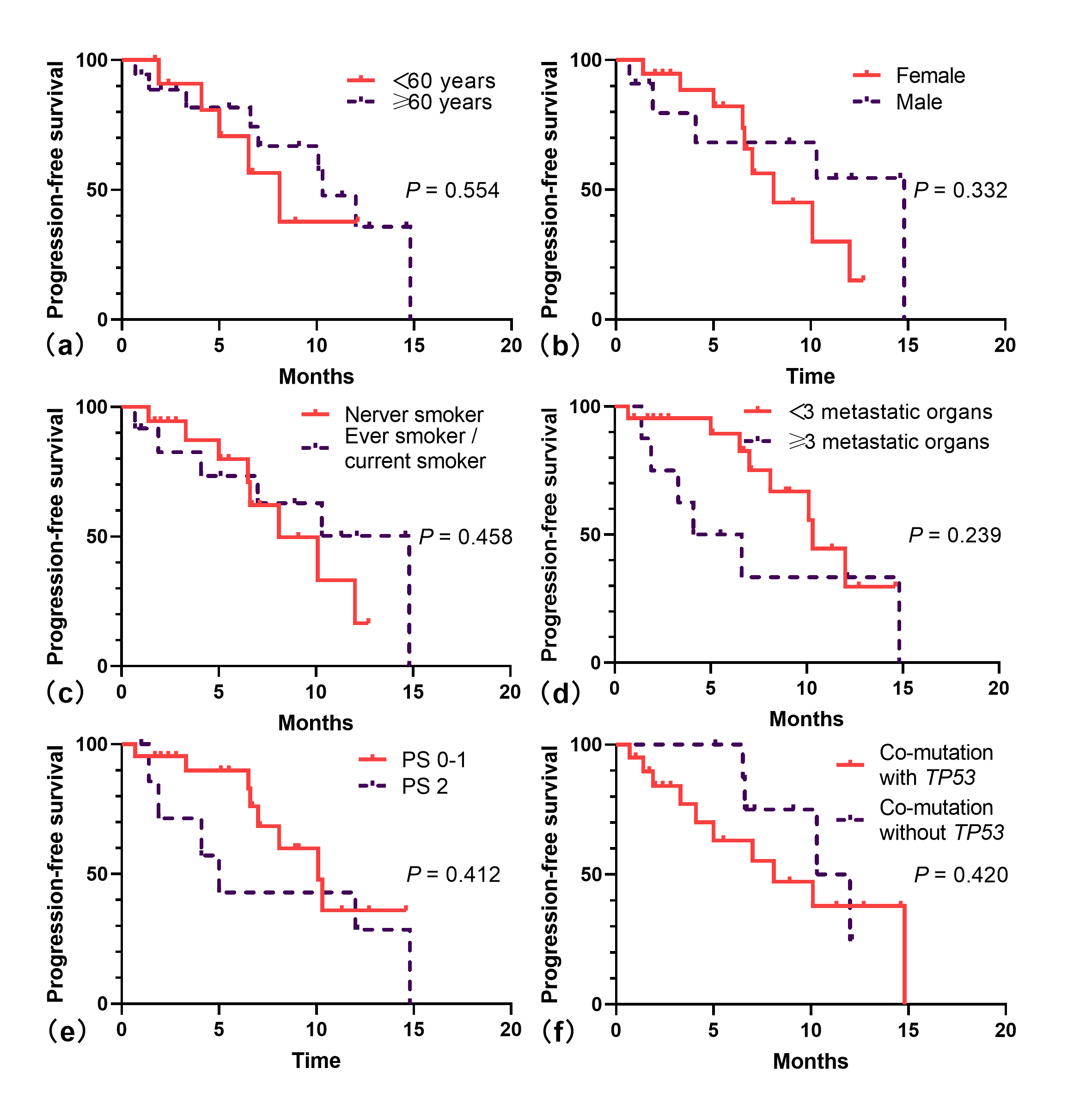

Supplement: Supplementary file 1 [file Image2.TIF]

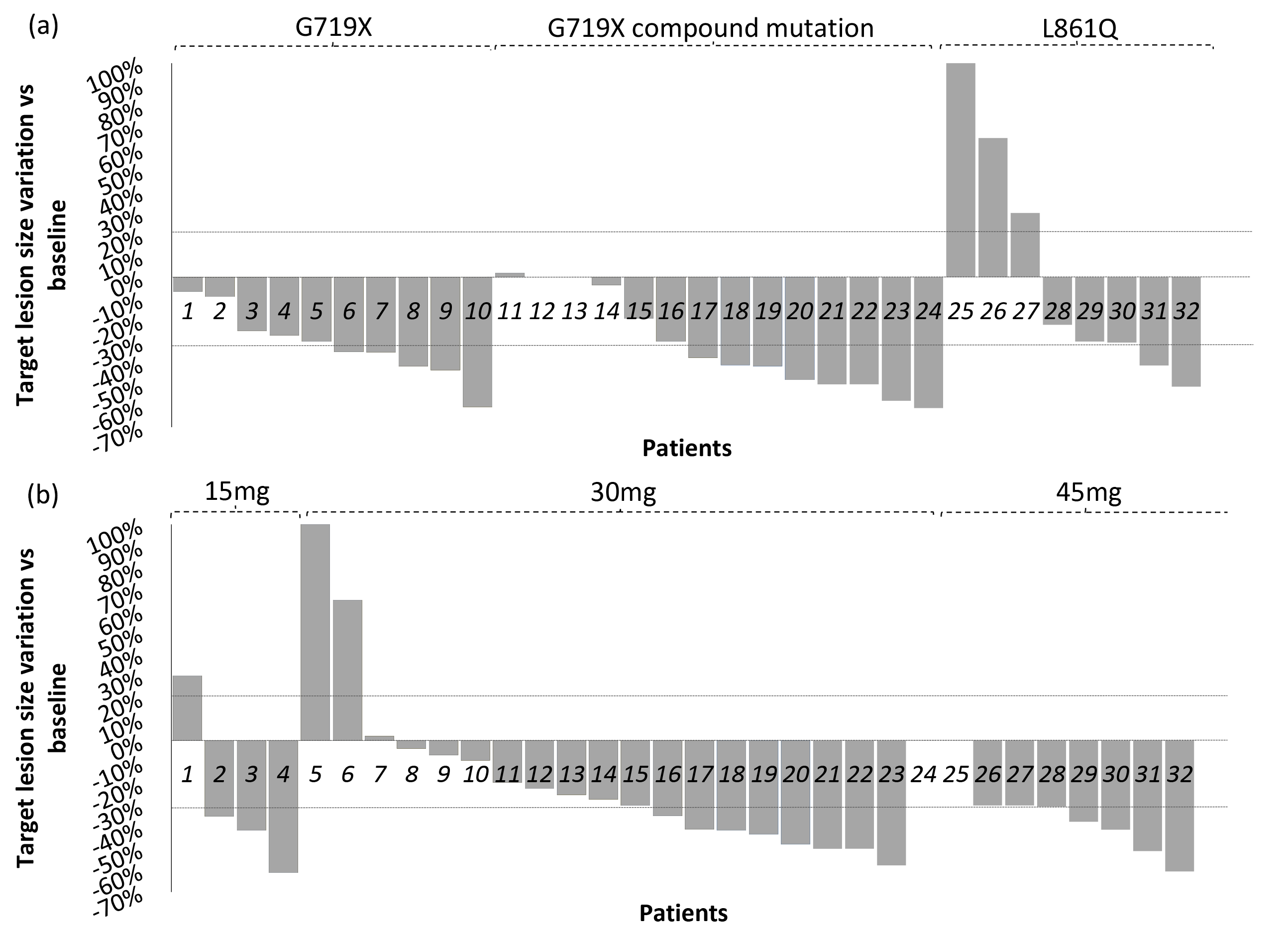

Supplement: Supplementary file 2 [file Image1.TIF]
